# Characteristics of distal radius fractures in east China-an observational cohort study of 1954 individual fractures

**DOI:** 10.1186/s12891-023-06742-x

**Published:** 2023-08-02

**Authors:** Zhenyu Luo, Wei Zhu, Chao Jiang, Wei He, Hua Zuo

**Affiliations:** Affiliated Hospital of Jiangsu University, Zhenjiang Jiangsu, China

**Keywords:** Distal radius fracture, Gender distribution, Age distribution, Osteoporosis, Seasonality

## Abstract

**Objective:**

To investigate the characteristics and seasonal patterns of distal radius fractures (DRFs) over the preceding five years, with the aim of establishing a clinical foundation for the prevention and management of such fractures within this region.

**Methods:**

Utilizing the Picture Archiving and Communication Systems (PACS), the clinical records of 1954 patients diagnosed with DRFs and admitted to the Affiliated Hospital of Jiangsu University between January 2017 and December 2021 were compiled. The analysis encompassed factors such as age, gender, visitation timing, fracture side, and presence of osteoporosis.

**Results:**

Out of the total 1954 distal radius fractures, 731 were males (37.4%) and the male to female ratio was 0.59:1. The median age of patients with DRFs was 56 years, with the 25th percentile being 38 years and the 75th percentile being 67 years. The average age was 50 years (standard deviation 23.3) and 1033 cases (52.7%) occurred on the left side, 885 cases (45.1%) on the right side, and 36 cases (1.8%) were bilateral, with the left side being the most frequently affected. The age group of 61–70 years (23.9%, 467/1954) exhibited the highest proportion, and the most prominent age group for males was 11–20 years (23.8%, 174/731), whereas for females it was 61–70 years (30.83%, 377/1223). In the 50 years and older group, there were 276 males and 991 females (ratio 1:3.59), with osteoporosis in 536 cases, accounting for 42.03% of the group. In terms of seasonal distribution, the highest incidence occurred during the summer and autumn months (55.1%, 1076/1954) and there were gender differences in different seasons.

**Conclusion:**

In east China, DRFs were predominantly female and left-sided, with the highest proportion in the age group of 61–70 years and in summer and autumn. Furthermore, gender differences were observed between the warm and cold seasons.

## Introduction

DRFs are a clinically common type of fracture characterized by low energy fractures occurring approximately proximal to the articular surface of the distal radius, accounting for approximately one sixth of total fractures [1, 2]. The incidence of DRFs peaks among the pediatric and geriatric populations, and with the increase of age, the incidence shows an increasing trend [3], which has garnered increasing attention among clinical workers. The management of DRF primarily involves non-surgical and surgical approaches, with the proportion of surgical interventions progressively rising over the years. ORIF (open reduction and internal fixation) with volar plating almost completely replaces external fixation stent and percutaneous needle fixation in the treatment of DRF [4]. Domestic and foreign literature reports [5–8], show that the incidence of DRF varies with age, season and lifestyle. The study offered a rigorous and valuable analysis of DRFs within a five-year span, especially seasonal patterns, drawing from a large patient dataset in East China by utilizing the PACS. This is an insightful exploration into demographic and temporal factors associated with DRFs, including age, gender, the side of fracture, and seasonal occurrence. The study also provides information about the prevalence of osteoporosis in relation to DRFs.

Overall, the research represents an essential step in understanding the multifactorial nature of DRFs in East China, and it opens avenues for developing focused prevention strategies and improving fracture management, thus enhancing patient outcomes.

## Data and methods

### Subjects

The complete dataset of patients diagnosed and treated for DRFs between January 1, 2017, and December 31, 2021, was thoroughly screened utilizing PACS at the Affiliated Hospital of Jiangsu University. PACS stands for Picture Archiving and Communication System and it is a medical imaging technology that is used to store, retrieve, manage, and distribute medical images and related patient information. The screened data included essential information such as name, gender, age, timing, side, and imaging diagnosis.

### Inclusion and exclusion criteria

#### Inclusion criteria

1. Radiographic diagnosis of DRF; 2. Initial visit.

#### Exclusion criteria

1. Patients with old DRF (the injury occurred beyond a duration of three weeks); 2. Patients with neoplastic pathologic fracture; 3. Patients reviewed after fracture.

### Methods

A comprehensive collection of 16,410 cases of DRFs was obtained, from which 1954 patients were screened based on predefined inclusion and exclusion criteria. Pertinent factors including age, gender, visitation timing, fracture side, and presence of osteoporosis were recorded for each patient. The collected data was subsequently analyzed to explore patterns and trends. Radiographs evaluate bone density by assessing radiolucency, trabecular architecture, and cortical thickness [9]. Furthermore, only cases with bone loss exceeding 30% can be identified as positive on plain X-rays [10].

### Statistical analysis

Statistical Package for the Social Sciences version 25.0 (SPSS 25.0 IBM) statistical software was used to analyze the data to observe the age and gender distribution of patients with DRF, as well as whether there were differences in gender structure and age distribution between summer, autumn and spring and winter. The statistical data were compared using a chi-square test, with a two-sided test level of α = 0.05.

## Results

### General information and gender distribution by age group

**Fig. 1:**
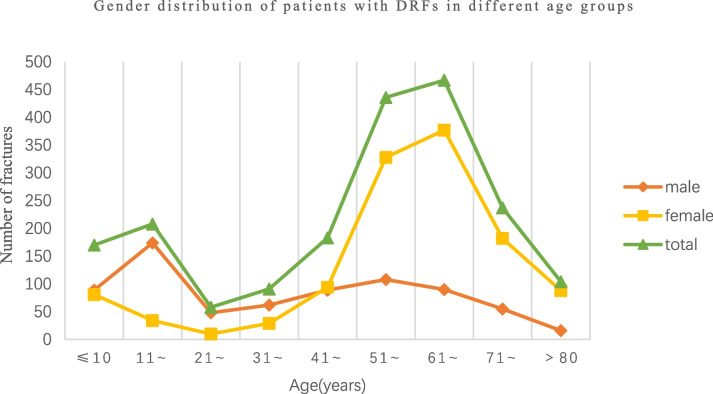
Gender distribution of patients with DRFs in different age groups

A total of 1954 patients with DRF were admitted to our hospital from January 2017 to December 2021. The median age of patients with DRFs was 56 years, with the 25th percentile being 38 years and the 75th percentile being 67 years. The average age was 50 years (standard deviation 23.3) and 1033 cases (52.7%) occurred on the left side, 885 cases (45.1%) on the right side, and 36 cases (1.8%) were bilateral. As is shown in Table 1, patients for surgery on DRF each year showed a slow increase. The gender distribution of each age group was shown in Table 2, and 731 cases were males (37.4%) and the male to female ratio was 0.59: 1. According to the age distribution, the highest proportion was observed in the 61–70 years age group, accounting for 23.90%. Among males, the highest proportion was found in the 11–20 years age group, accounting for 23.80% (174/731). Among females, the highest proportion was observed in the 61–70 years age group, accounting for 30.83% (377/1223). When the control group was defined as the age group of 61–70 years, the test level α' = α⁄ (2 (k-1)) = 0.003, where k represents the number of age groups. Statistical analysis revealed a significant gender difference between each age group below 50 years and the control group (*P* < 0.003). However, no significant gender difference was observed between the age group above 50 years and the control group (*P* > 0.003). Figure 1 illustrates a bimodal age distribution among patients with DRFs, indicating that the highest proportion of patients falls within two age groups: 11–20 years and 61–70 years. Among women, the highest proportion is observed in the 61–70 age range, while among men, it is highest in the 11–20 age range.

**Table 1:** The proportion of patients with DRFs undergoing surgery from 2017 to 2021 (Cases)

Year	Surgical patient	Total patient	Constituent ratio (%)
2017	67	374	17.91
2018	81	436	18.58
2019	76	401	18.95
2020	58	301	19.27
2021	90	442	20.36
Total	372	1954	19.04

**Table 2:** Distribution of patients with DRFs in different age groups [Cases (%)]

Age group	male	female	Total
≤ 10	89	81	170 (8.7)
11 ~	174	34	208 (10.64)
21 ~	48	10	58 (2.97)
31 ~	62	29	91 (4.66)
41 ~	89	94	183 (9.37)
51 ~	108	328	436 (22.31)
61 ~	90	377	467 (23.90)
71 ~	55	182	237 (12.13)
> 80	16	88	104 (5.32)
Total	731	1223	1954 (100)

### Age, sex and osteoporosis of middle-aged and elderly patients

The analysis of 1267 middle-aged and elderly patients after the age of 50 showed that there were 276 males and 991 females (male to female ratio 1:3.59). The plain X-rays showed that there were 536 cases of osteoporosis, accounting for 42.03% of the middle-aged and elderly patients with fracture If dual-energy X-ray absorptiometry (DXA) examinations were conducted, the proportion would be even higher. However, the further examination of bone mineral density measurement was only 7.97% (101/1267), and patients with the anti-osteoporosis treatment after fracture, such as bisphosphonates, active vitamin D and its analogues, was 21.07% (267/1267). Our findings indicate that after the age of 55, the proportion of women patients is more than three times that of men, and the osteoporosis rate in the age group of 65–69 reaches 47.11%. Furthermore, as age increases, the osteoporosis rate also rises, with nearly all patients experiencing osteoporosis after the age of 90., as shown in Table 3.

**Table 3:** Distribution of osteoporosis rate in middle-aged and elderly patients with DRFs by gender and age group (Cases)

Age Group (years)	Gender		Sex ratio	Total	Osteoporosis	Osteoporosis ratio (%)
	Male	Female				
50 ~ 54	61	124	1: 2.03	185	31	16.76
55 ~ 59	46	191	1: 4.15	237	56	23.63
60 ~ 64	50	187	1: 3.74	237	85	35.86
65 ~ 69	42	200	1: 4.76	242	114	47.11
70 ~ 74	29	101	1: 3.48	130	78	60.00
75 ~ 79	24	86	1: 3.58	110	74	67.27
80 ~ 84	16	61	1: 3.81	77	57	74.03
85 ~ 89	4	33	1: 8.25	37	30	81.08
90 ~	4	8	1: 2	12	11	91.67
Total	276	991	1: 3.59	1267	536	42.30

### Data analysis conducted on patients with DRFs in different seasons

**Fig. 2:**
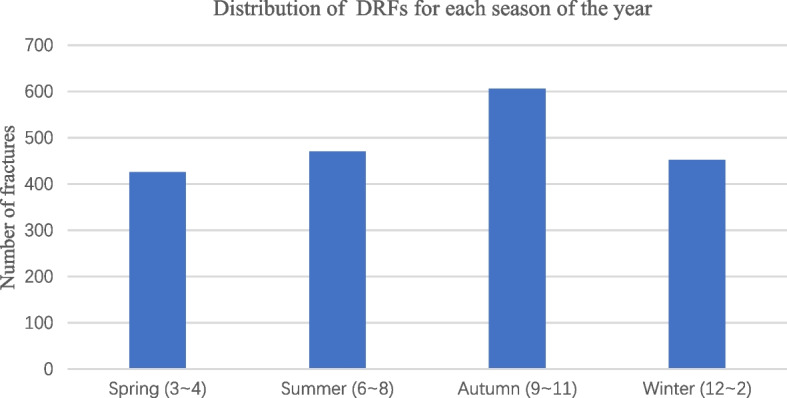
Distribution of DRFs for each season of the year

When grouping all patients based on the timing of their visits, it was observed that distal radius fractures were more prevalent during the warmer seasons, namely autumn and summer (Fig. 2). Upon dividing the patients into two groups based on the warm season and the cold season (Table 4), a statistically significant gender difference was observed between the two groups (χ2 = 6.663, *P* < 0.05). Specifically, the gender difference was statistically significant only in the 41–50 years age group (*P* < 0.05), while the other age groups did not exhibit a statistically significant gender difference (*P* > 0.05). Furthermore, the highest composition ratio in both groups was observed among individuals aged 61–70 years. And in the male group, the highest composition ratio was found in the 11–20 years age group, while in the female group, the highest composition ratio was observed in the 61–70 years age group.

**Table 4:** Comparison of age and gender composition of patients in different seasons [cases (%)]

Age group (years)	Group A (June to November)			Group B (December to May)			Chi square value	*P* values
	Male	Female	Total	Male	Female	Total		
< 10	64 (14.88)	48 (7.43)	112 (10.41)	25 (8.31)	33 (5.72)	58 (6.61)	3.019	0.082
11 ~ 20	96 (22.33)	18 (2.79)	114 (10.59)	78 (25.91)	16 (2.77)	94 (10.71)	0.057	0.811
21 ~ 30	31 (7.21)	8 (1.24)	39 (3.62)	17 (5.65)	2 (0.35)	19 (2.16)	0.893	0.345
31 ~ 40	35 (8.14)	21 (3.25)	56 (5.20)	27 (8.97)	8 (1.39)	35 (3.99)	2.127	0.145
41 ~ 50	49 (11.40)	38 (5.88)	87 (8.09)	40 (13.29)	56 (9.71)	96 (10.93)	3.924	0.048
51 ~ 60	63 (14.65)	170 (26.32)	233 (21.65)	45 (14.95)	158 (27.38)	203 (23.12)	1.381	0.240
61 ~ 70	47 (10.93)	198 (30.65)	245 (22.77)	43 (14.29)	179 (31.02)	222 (25.28)	0.003	0.959
71 ~ 80	33 (7.67)	90 (13.93)	123 (11.43)	22 (7.31)	92 (15.94)	114 (12.98)	1.883	0.170
> 80	12 (2.79)	55 (8.51)	67 (6.23)	4 (1.33)	33 (5.72)	37 (4.21)	0.923	0.337
Total	430 (100)	646 (100)	1076 (100)	301 (100)	577 (100)	878 (100)	6.663	0.010

## Discussion

### General overview of DRFs and gender distribution across various age groups

This study revealed an annual surgical rate of approximately 19% for distal radius fractures, with a gradual upward trend. Currently, closed reduction and plaster fixation remain the predominant treatment modalities for the majority of DRFs. An epidemiological study in Finland [11] showed that a range of 15% to 18% of DRFs underwent surgical intervention, and the annual incidence of surgical procedures remained relatively stable. In the population aged 0–17 years in Sweden, the incidence of distal radius fractures was found to be higher among male patients compared to female patients [12], possibly due to the fact that the higher participation of males in high-energy physical activities compared to females; And a higher proportion of patients within this age group were male as compared to female in our study. Rundgrn et al. [5] found that DRFs exhibit a significant disparity in gender distribution, with a noticeable increase in incidence among women after the age of 50, which is comparatively slower in men. It can be attributed to the decline in estrogen levels and bone mass in women after menopause, resulting in a higher fracture incidence compared to men [13]. Jantzen et al. [14] found that the prevalence of osteoporosis in Colles fractures was as high as 50.3% in women and 27% in men. This observation leads to a clear conclusion that the occurrence of DRFs is significantly higher in middle-aged and elderly women compared to men, which is directly associated with the occurrence of osteoporosis. The latest study [15] shows that the prevalence of osteoporosis in Chinese men remains relatively stable before the age of 75. Therefore, in this study, the increase in DRFs among men of all ages after the age of 50 exhibited a comparatively stable trend. Karl et al. [16] found that the incidence of DRFs displayed a bimodal distribution, with the highest rates observed in the age groups under 18 and over 65. Raudasoja et al. [11] showed that DRFs peaked in the pediatric population and older women, where the incidence was more than four times higher compared to men of the same age group. Both children and the elderly also exhibited the highest proportion among patients with DRFs in our study.

### Data analysis of middle-aged and elderly patients with DRFs

The rising prevalence of osteoporosis has contributed to an increase in fragility fractures of the upper extremity, which are characterized by occurring due to low-energy trauma, such as falls from a standing height or lower. [17]. DXA is considered the gold standard of methods used to diagnose osteoporosis [18]. In our study, we only screened patients with radiographic evidence of osteoporosis, which may result in an underestimation of the actual number of patients with osteoporosis. This study suggests that over 40% of patients aged 50 and above with DRFs have osteoporosis, but merely 21.07% of patients received anti-osteoporosis therapy. Bougioukli et al. [19] found that individuals who suffer from fragility fractures are not receiving sufficient osteoporosis treatment. An epidemiological study in Hong Kong [20] suggests that nearly half of all secondary fractures occur within a two-year timeframe following the initial significant fragility fracture and this period represents a crucial opportunity for osteoporosis treatment and fall prevention. Freyschuss et al. [21] found that out of Swedish patients aged 50 and above who experienced fragility fractures, only 10% received anti-resorptive treatment within one year of their initial fragility fracture. In France, Fardellone et al. [22] found that after the initiation of anti-resorptive therapy, the incidence of subsequent fractures was reduced by 60%. Therefore, we strongly advocate for the implementation of regular anti-osteoporosis treatment, such as the administration of bisphosphonates, following the occurrence of a fragility fracture. Additionally, meticulous monitoring of the treatment progress, encompassing both effectiveness and adverse reactions, is equally important. To reduce the risk of subsequent fractures in the elderly, DXA is recommended for bone mineral density measurement in women ≥ 65 years old and men ≥ 70 years old to identify osteoporosis earlier. Simultaneously, it is crucial to prioritize the assessment and intervention of fall-related risk factors in elderly individuals with osteoporosis, so as to reduce the occurrence of fractures, identify high-risk populations, and initiate early prevention and treatment of osteoporosis. The utilization of calcium and/or vitamin D supplementation is recommended in conjunction with anti-osteoporosis medications [23]. Additionally, it is imperative to enhance osteoporosis education. In daily life, the elderly should prioritize fall prevention measures such as being accompanied by family members when going out. And attention should be given to personal dietary habits, including consuming a balanced diet rich in calcium, low in salt, and with adequate protein [24]. Adopting healthy lifestyle habits, such as quitting smoking, limiting alcohol consumption, and increasing outdoor activities for exposure to sunlight, are also essential. Furthermore, engaging in appropriate functional exercises to enhance muscle strength can be beneficial. These measures have a certain significance.

### Seasonal variation of DRFs in east China

The survey indicated that DRFs were more common during warm seasons (summer and autumn). However, Warrender WJ [25] found that the incidence of DRFs is significantly higher during the winter season compared to other seasons, likely due to the presence of slippery road surfaces during winter months. Johnson NA et al. [26] also found that in cold weather, the incidence of DRFs was significantly increased. Ogliari et al. [27] found that the frequency of frost days directly correlates with the occurrence of ulna, radius and humerus fractures during the winter season. Interestingly, a contrasting pattern emerges for patients in our study. Stotz A et al. [28] showed that high ambient temperatures decrease blood pressure (BP) in young and middle-aged adults and may result in orthostatic hypotension, increasing the risk of falls in older adults. Additionally, the higher incidence of outdoor exercise during the summer and reduced activities during the winter may contribute to the phenomenon. Furthermore, the prevalence of slippery roads during the rainy season, which occurs more frequently in summer and autumn in East China, could also be a contributing factor. The reasons for the difference in gender composition across seasons within this study has not been conclusively established. One possible explanation could be the difference in physical activities between men and women in different seasons Additionally, occupational differences between genders could be another contributing factor. Men and women might have different tendencies in their choice of activities, hobbies, or occupations based on societal expectations or personal preferences. Various factors, both unchangeable (age, gender, race, and ethnicity) and modifiable (behavioral and personality characteristics, environmental circumstances and community settings), can have an impact on the motivation and maintenance of physical activity among adults [29]. Hence, it would be valuable to investigate the influence of environmental factors on the gender difference in DRFs. However, these are preliminary ideas, and further research is required to provide a conclusive answer.

Clinically, the findings could refine healthcare practices by enabling the early identification of at-risk individuals and guiding appropriate interventions. The data point towards an increased prevalence among females aged between 61–70 years, particularly during summer and autumn. Education on fall prevention aimed at older women, especially during the high-risk seasons, may reduce DRFs incidence. The observed seasonal patterns could also inform the allocation of healthcare resources to anticipate and manage peak fracture occurrences. Additionally, given the noted correlation with osteoporosis, particularly among patients over 50, osteoporosis management strategies may prove instrumental in fracture prevention.

#### Limitations of this study

1. This study was a retrospective analysis, without reference to etiology, fracture type and treatment plan; 2. This is a single-center study, and did not make statistics on multiple hospitals in the whole east China; 3. Some patients who had their own radiographs were not included in the data collection process, resulting in certain data bias.

In conclusion, this study suggests that the epidemiological characteristics of DRFs in east China from 2017 to 2021 are as follows: the patient cohort exhibited a bimodal age distribution, with a predominant presence of females and left-sided fractures. Specifically, the age group of 61–70 years exhibited the highest proportion, and the most prominent age group for males was 11–20 years, whereas for females it was 61–70 years. Among middle-aged and elderly individuals aged 50 years and above, a substantial proportion, approaching nearly half, exhibited the presence of osteoporosis. With advancing age, the rate of osteoporosis also increased. The occurrence of distal radius fractures in this region exhibits notable seasonality, with a higher frequency observed during the summer and autumn seasons. Further investigation is required to explore potential variations in gender composition across different seasons. In the prevention of DRF, the primary focus lies in preventing low-energy fragility fractures among middle-aged and elderly individuals. This involves enhancing public education on osteoporosis, emphasizing early diagnosis and intervention, and promoting improvements in dietary and lifestyle habits to reduce the occurrence of osteoporosis. Additionally, it is crucial to intensify anti-osteoporosis treatment for the elderly population following a fracture.

## Data Availability

The datasets used and/or analyzed during the current study are available from the corresponding author on reasonable request.
